# Socioeconomic inequalities in children’s weight, height and BMI trajectories in Norway

**DOI:** 10.1038/s41598-021-84615-w

**Published:** 2021-03-02

**Authors:** Teferi Mekonnen, Eleni Papadopoulou, Onyebuchi A. Arah, Anne Lise Brantsæter, Nanna Lien, Mekdes K. Gebremariam

**Affiliations:** 1 Department of Nutrition, Faculty of Medicine, Institute of Basic Medical Sciences, University of Oslo, Oslo, Norway; 2 Section of Environmental Exposure and Epidemiology, Norwegian Institute of Public Health, Oslo, Norway; 3 Department of Epidemiology and Department of Statistics, University of California, Los Angeles (UCLA), Los Angeles, USA

**Keywords:** Risk factors, Epidemiology

## Abstract

Studies exploring when social inequalities in body mass index (BMI) and its composites emerge and how these evolve with age are limited. Thus, this study explored parental income and education related inequalities in children’s weight, height, weight velocity and body mass index among Norwegian children from 1 month to 8 years. The study population included 59,927 family/children pairs participating in the Norwegian Mother, Father, and Child Cohort Study. Growth was modelled using the Jenss–Bayley model and linear mixed effects analyses were conducted. Maternal and paternal educational differences in children’s weight and BMI trajectories emerged during infancy, continuing to age 8 years. Parental income-related inequalities in children’s weight were observed from the age of 1 month to 4 years for maternal and up to 1 year for paternal income-related differences but then disappeared. Parental income-related inequalities in child’s BMI were observed from 18 months to 8 years for maternal income, and from 9 months to 8 years for paternal income-related differences. These results suggest that social inequalities in children’s BMI present early in infancy and continue to 8 years of age. The inequalities sometimes differed by indicator of socioeconomic position used. Interventions to combat these inequalities early in life are, thus needed.

## Introduction

Socioeconomic inequalities in overweight (OW)/obesity (OB) among children and adolescents are prevalent^1–3^ and represent important public health concerns. Tackling these inequalities is a key public health goal because of the short and long-term adverse health effects of early life OW and OB^4,5^, including the tracking of body weight from childhood into adolescence^6^ and adulthood^7–11^. Socioeconomic inequalities in children’s BMI trajectories can vary with children’s age because of the socioeconomic gradients in various perinatal and early life factors, including breastfeeding^12^, complementary feeding practices^13,14^, childhood dietary behaviours^15–18^ and sedentary behaviours^19^. Tackling such inequalities requires an understanding of when in early life they emerge and how they evolve with increasing age.

Few studies with available longitudinal data from birth to childhood have explored how early socioeconomic inequalities in body mass index (BMI) trajectories emerge and how they evolve throughout childhood. Evidence from these studies revealed mixed results regarding the timing at which distinct BMI trajectories by socioeconomic status emerge^3,20–26^. Therefore, there is a need for more longitudinal studies with data from birth and with long periods of follow-up. Additionally, BMI changes could be attributable to changes in weight, height or both. Few studies with data from birth to childhood have considered weight and height measurements along with BMI^20,24^. Thus, exploring inequalities in children’s weight and height trajectories in addition to BMI could help to understand the roots of the inequalities in BMI or OW/OB among children. Another important aspect that helps to better explore the origin of inequalities in children’s BMI could be exploring socioeconomic inequalities in the rate of child growth per unit of time (e.g. weight gain velocities). In this regard, there is a lack of studies assessing inequalities in weight gain velocities.

In addition, it is important to take into account different indicators of socioeconomic position (SEP) while assessing socioeconomic inequalities in OW/OB among children, as different SEP indicators might influence the pathways leading to health inequalities in different manners. For example, income could influence the choice of healthy vs unhealthy lifestyle behaviors through the ability to afford healthier food alternatives. On the other hand, education could influence food choice by equipping parents with skills to gain better access to health information and to critically evaluate this information^27^. Most studies have used different SEP indicators interchangeably^1,21,26,28^. However, studies are showing the independent influence on anthropometric markers exerted by income and education. For instance, studies among adults in the US demonstrated that BMI and skinfold thickness among women were independently associated with education, but not with income; among men these anthropometric markers were independently associated with income only^29,30^. Another study among Brazilian adults showed that in less developed regions obesity among women was associated with both income and education, but in more economically developed regions, obesity among women was associated only with education. Among men, obesity was positively associated with income and to a certain extent with education^31^. In addition, limited evidence of a potential interaction between SEP indicators (i.e. education and income) on its association with OW/OB exists^32^.

Therefore, this study aimed to examine the associations between various measures of SEP and growth trajectories (i.e. BMI, weight, weight gain velocity, and height) among Norwegian children from birth to 8 years of age using data from one of the largest prospective pregnancy cohorts in the world.

## Methods

### Study design and sample

The Norwegian Mother, Father and Child Cohort Study (MoBa) is a population-based pregnancy cohort study conducted by the Norwegian Institute of Public Health^33^. Participants were recruited from all over Norway from 1999 to 2008. The invitation to participate was sent to pregnant women prior to the first ultrasound examination offered to all free of charge. The women consented to participation in 41% of the pregnancies. The cohort now includes 114,500 children, 95,200 mothers and 75,200 fathers. Included women received questionnaires at 17, 22 and 30 weeks of pregnancy, and when the child was 6 months, 18 months, 3 years, 5 years, 7 years and 8 years old. The current study is based on version 12 of the quality-assured data files released for research in January 2019, with linkage to the Medical Birth Registry of Norway, the national health registry containing information about all births in Norway. The establishment of MoBa and the initial data collection was based on a license from the Norwegian Data Protection Agency and approval from The Regional Committees for Medical and Health Research Ethics. The MoBa cohort is now based on regulations related to the Norwegian Health Registry Act. The current study was approved by the Regional Committees for Medical and Health Research Ethics (REK 2018/470). Written informed consent was obtained from each MoBa participant upon recruitment. All experiments were performed in accordance with relevant guidelines and regulations.

After exclusion of women with multiple gestations, malformations and chromosomal abnormalities, 83,475 mothers with live-born singleton births remained. Of these, 70,913 had complete data on maternal and paternal educational level and income status. Further exclusions were made based on missing data on birth weight and length, missing postnatal weight or height/length measurements, or missing data for the included covariates, yielding a final sample of 59,927 out of the 83,475 (71.8%). Supplementary Figure S1 shows the inclusion chart of the participants.

### Measures

Parental socioeconomic position was measured by self-reported maternal and paternal education and income during pregnancy (baseline questionnaire). Maternal and paternal educational status was defined into three categories as low (≤ 12 years of education), medium (13–16 years of education) and high (≥ 17 years of education). We have used average household income per year done in accordance with data from the statistics Norway at the time of baseline data collection (1999–2008) to define income status. Maternal and paternal income were defined as earned “below mean income” if their annual income is < 300,000 NOK per year and earned “greater than/equal to mean income” if their annual income is ≥ 300,000 NOK per year.

Anthropometric measurements of children in Norway are routinely conducted by health professionals at well child clinics (i.e. up to 5 years) and at school health services (i.e. after 5 years) according to guidelines from the Norwegian directorate of health^34^.

Mothers reported the weight and length/height of their child at 11 age points; 6 weeks, 3 months, 6 months, 8 months, 1 year, 1.5 years, 2 years, 3 years, 5 years, 7 years, 8 years (details on anthropometric measurements described in Supplementary Table S1). Birth weight and length data were obtained from the Medical birth registry of Norway. From 6 weeks to 18 months, mothers were asked to refer to their children’s health card, while no specification was provided for measurements from 2 to 8 years.

Implausible anthropometric values were identified and excluded by separately implementing two different methods; (1) by comparing with the WHO Growth standards, as a weight-for-age or height-for-age z-score < 6 SD below or > 6 SD (5 SD for weight) above the mean^35^, and (2) by identifying measured values with a > 3 SD and <  − 3SD difference from the predicted value as derived from the Jenss–Bayley growth curve model. After exclusion of implausible values, 498,393 and 488,334 repeated measurements of weight and length/height were available for our study population.

Age and gender standardized BMI (in kg/m^2^), weight (in grams (g)), weight gain velocities (in g/month) and height (in centimeters (cm)) growth trajectories were the outcome variables. Individual growth trajectories for weight and length/height were obtained by modelling the overall growth from the age of 1 month to 8 years using the Jenss-Bayley growth curve model^36^. A mixed-effect approach using the stochastic approximation of expectation–maximization (SAEM) algorithm were applied to assess individual growth trajectories^37^. Then, weight, weight gain velocities, and length/height, BMI (kg/m^2^) at 14 age points (1 month, 2 months, 3 months, 6 months, 9 months, 12 months and 18 months, 2 years, 3 years, 4 years, 5 years, 6 years, 7 years and 8 years) were calculated using the growth model derivatives. Given the high correlation between the measured and predicted height in the different age points (concordance correlation coefficients range ρ_c_ = 0.77–0.97) and weight (concordance correlation coefficients range ρ_c_ = 0.81–0.98) (Supplementary Table S2), the outcome variables used in this study were based on the predicted anthropometrics derived by using the growth model derivatives. Childhood weight status (not overweight/obese, overweight/obese) at the age 2 to 8 years was defined by applying the age and gender specific International Obesity Task Force BMI cut-offs (kg/m^2^)^38^ in the predicted BMI values.

### Statistical analysis

Graphical illustration of the overall distributions of children’s anthropometric data (i.e. Weight, weight gain velocity, height, and BMI) at the ages of 1 month to 8 years at 14-time points was created. Linear mixed effect models with random intercept at the child-level and a random slope for age were used to analyze associations between SEP indicators and children’s anthropometry at 14 time points between ages of 1 month to 8 years. The associations between SEP indicators and children’s anthropometry at 14 time points were illustrated using the high SEP position as a reference. The average marginal effects of SEP indicators on children’s anthropometry at 14 time points were estimated. Then, the estimated marginal effects were presented in tables and graphs. The other SEP indicator was mutually adjusted for in the analyses in order to estimate the independent effect of each SEP indicator (parental education, parental income). The interaction between the SEP indicators was tested. Adjustment for child gender, birth weight, parity, maternal civil status, and gestational age was made. Multicollinearity was checked. The main analyses were performed with Stata version 16 statistical software (Stata Corporation, College Station, Texas, USA). R version 3.2.2^39,40^ was used for the growth models. The p-value for statistical significance was set to *P* < 0.05.

## Results

Among 59,927 families /child pairs included in the study, 30.3% and 63.6% of the children had mothers with low education and who earned below mean income, respectively. The proportion of children with low paternal education and fathers who earned below mean income were 45.4% and 34.2%, respectively (Table 1).

**Table 1:** Socio-demographic characteristics of family/child pairs included in this study (N = 59,927).

Socio-demographic variables	N (%)
**Child gender**	
Male	30,738 (51.3)
Female	29,189 (48.7)
**Maternal marital status**	
Living with partner	58,468 (97.6)
Others	1459 (2.4)
**Parity**	
Primiparaous	27,610 (46.1)
Multiparous	32,317 (53.9)
Maternal educational status*	
Low	18,149 (30.3)
Medium	25,758 (43.0)
High	16,020 (26.7)
Paternal educational status*	
Low	27,197 (45.4)
Medium	17,407 (29.1)
High	15,323 (25.6)
Maternal income**	
Below mean income	31,129 (63.6)
Greater than/equal to mean income	21,798 (36.4)
Paternal income**	
Below mean income	20,504 (34.2)
Greater than/equal to mean income	39,423 (65.8)

*Low: completed ≤ 12 years of education, medium completed 13–16 years of education, and high completed ≥ 17 years of education.

**Below mean income < 300,000 Norwegian Krone per year and greater than/equal to mean income ≥ 300,000 Norwegian Krone per year.

The percentage of those with OW/OB among children aged 2 years to 8 years was higher for the lowest socioeconomic groups for all the SEP indicators used (Supplementary Table S3). The overall shape and distribution of children’s weight, weight gain velocity, height and BMI from age 1 month to 8 years (14-time points) is shown in the violin plot (Supplementary Fig. S2).

### SEP and child weight and weight gain velocity up to 8 years

**Figure 1:**
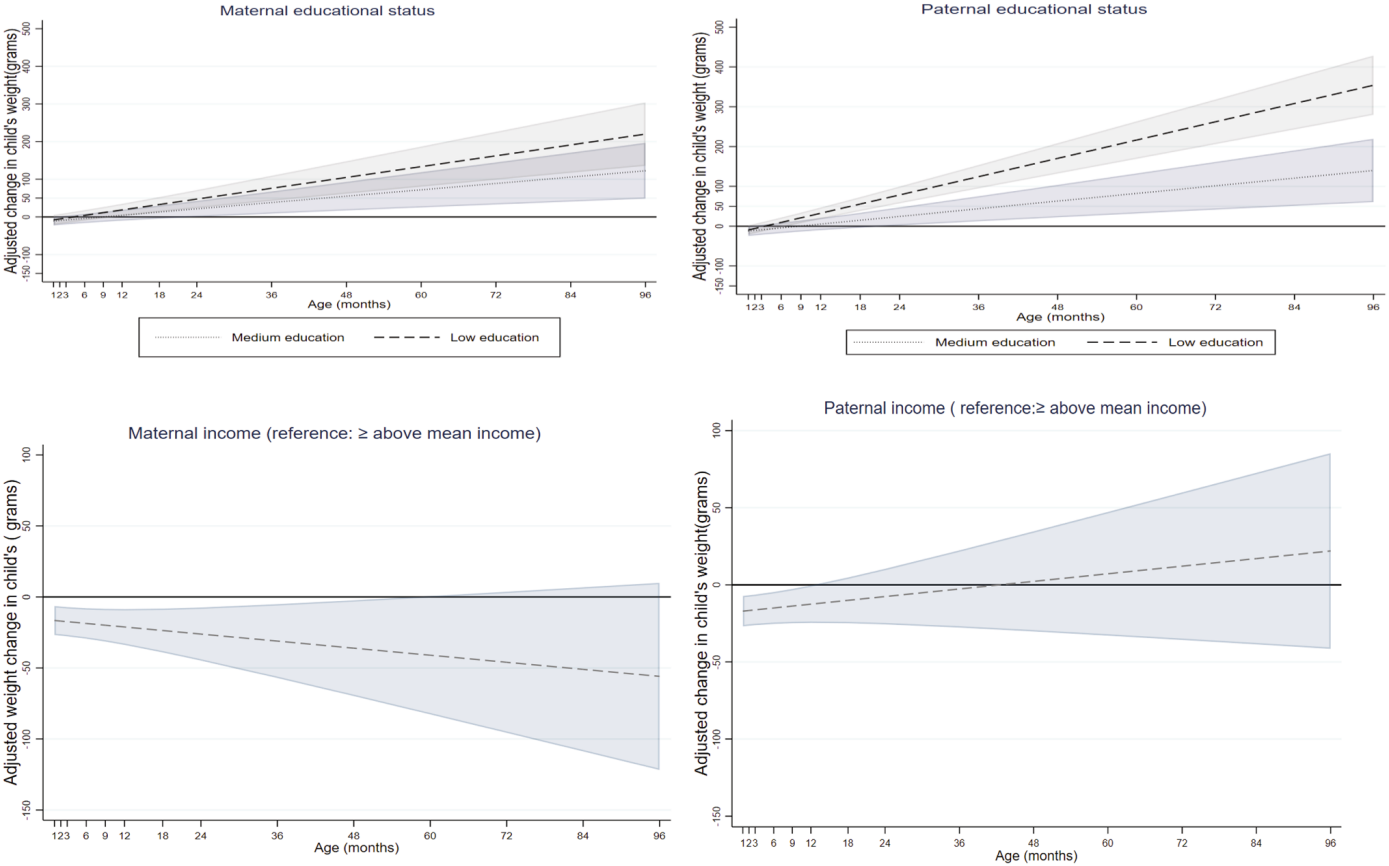
Associations between maternal and paternal education and income with children’s weight in grams from 1 month to 8 year (β coefficients in dash and dot lines and 95% CIs in solid lines).

Maternal educational level was inversely associated with children’s weight from the age of 1 year onwards (Fig. 1). Children of low educated mothers on average weighed more from the age of 1 year onwards compared to their peers with high educated mothers (e.g. weighed; 18.6 g more (95% CI 2.4, 34.7 g) at the age of 1 year and 219.9 g more (95% CI 135.8, 304.0 g) at the age of 8 years). Children of medium educated mothers weighed more from the age of 2 years to 8 years compared to their peers with high-educated mothers (Fig. 1, Supplementary Table S4).

Paternal educational level was inversely associated with children’s weight from the age of 9 months onwards (Fig. 1). Children of low educated fathers on average weighed more from the age of 9 months onwards compared to their peers with high-educated fathers (e.g. weighed; 21.1 g more (95% CI 8.0, 34.1 g) at the age of 9 months and 354.1 g more (95% CI 279.8, 428.4 g) at 8 years). Children of fathers with medium educational level on average weighed more from the age of 2 years to 8 years compared to their peers with high educated-fathers (Fig. 1, Supplementary Table S4).

Maternal income was positively associated with children’s weight from the age of 1 month up to 4 years (Fig. 1). Children of mothers who earned below mean income on average weighed less from the age of 1 month up to 4 years, compared to their peers with mothers who earned greater than/equal to mean income (e.g. weighed 16.6 g less (95% CI − 26.8, − 6.4) and 36.1 g less (95% CI − 69.8, − 2.3 g) at the age of 1 month and 4 years, respectively). This association was not present from the age of 5 years onwards (Fig. 1, Supplementary Table S4).

Paternal income was positively associated with children’s weight from 1 month to 1 year; after 1 year, the paternal income gradient in children’s weight was no longer significant (Fig. 1). Children of fathers who earned below mean income on average weighed less from the age of 1 month to 1 year, compared to their peers with fathers who earned greater than/equal to mean income (e.g. weighed 17.1 g less (95% CI − 26.9, − 7.2 g) at the age of 1 month and 12.5 g less 95% CI − 24.7, − 0.3 g) at 1 year age) (Fig. 1, Supplementary Table S4).

Children of low educated mothers on average had higher weight gain velocity from the age of 18 months to 6 years compared to their peers with high-educated mothers (e.g. gain 1.8 g/month more (95% CI 0.1, 3.5 g/month) at the age of 18 months and 2.5 g/month more (95% CI 0.0, 4.8 g/month) at age 6 years). No association was observed up to 18 months and after 7 years. Children of low educated fathers on average had higher weight gain velocity from 1 month to 8 years compared to their peers with high-educated fathers (e.g. gain 3.2 g/month more (95% CI 1.2, 5.1 g/month) at the age of 1 month and 4.3 g/month more 95% CI 1.3, 7.3 g/month) at age 8 years). Parental income was not associated with children’s weight gain velocity at any of the age points assessed (Supplementary Table S4).

### SEP and height among children aged up to 8 years

Maternal and paternal educational level were not associated with children’s height at any of the age points assessed (Supplementary Table S4).

**Figure 2:**
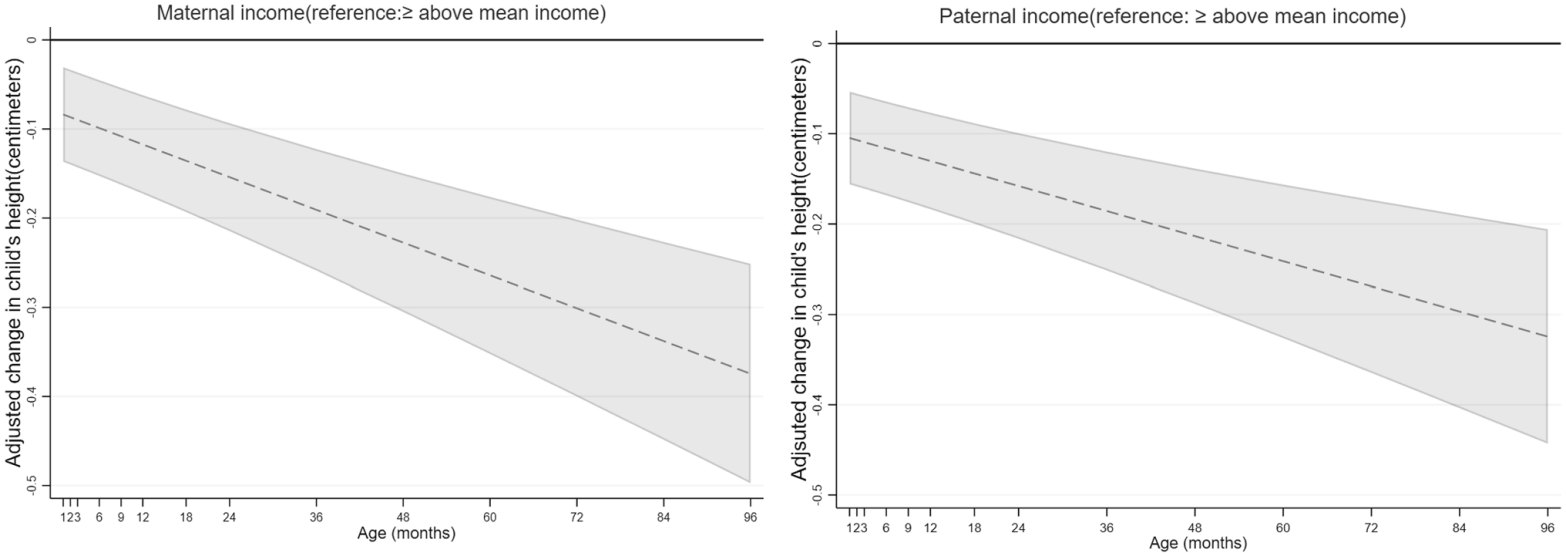
Associations between maternal and paternal income with children’s height in centimeters from 1 month to 8 year (β coefficients in dash lines and 95% CIs in solid lines).

Maternal income was positively associated with children’s height at the age of 1 month to 8 years (Fig. 2). Children of mothers who earned below mean income on average were shorter from the age of 1 month to 8 years compared to their peers with mothers who earned greater than /equal to mean income (e.g. 0.08 cm shorter (95% CI − 0.14, − 0.03 cm) at the age of 1 month and 0.37 cm shorter (95% CI − 0.50, − 0.25 cm) at the age of 8 years (Fig. 2, Supplementary Table S4).

Paternal income was positively associated with children’s height from the age of the 1 month to 8 years (Fig. 2). Children of fathers who earned below mean income on average were shorter from the age of the 1 month to 8 years compared to their peers with fathers who earned greater than /equal to mean income (e.g. 0.10 cm shorter (95% CI − 0.16, − 0.05 cm) at the 1st month and 0.32 cm shorter (95% CI − 0.44, − 0.21 cm) at 8 years (Fig. 2, Supplementary Table S4).

### SEP and BMI among children up to 8 years

**Figure 3:**
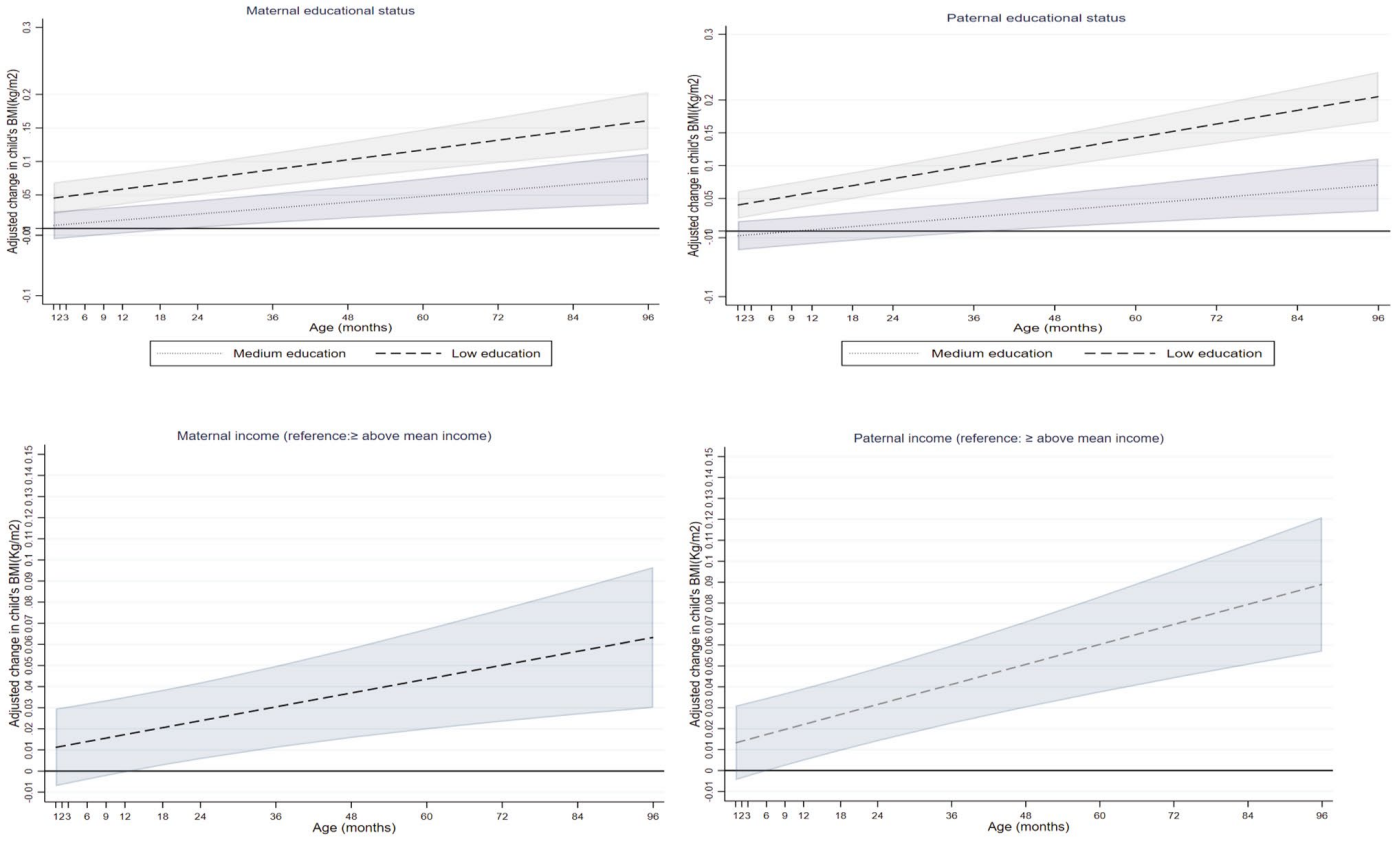
Associations between maternal and paternal education and income with children’s BMI from 1 month to 8 year (β coefficients in dash and dot lines and 95% CIs in solid lines).

Maternal educational level was inversely associated with children’s BMI (kg/m^2^) from the age of 1 month to 8 years (Fig. 3). Children of low educated mothers on average had a higher BMI from the age of 1 month to 8 years compared to their peers with mothers with high education (e.g. 0.05 kg/m^2^ higher (95% CI 0.02, 0.07 kg/m^2^) at the 1st month and 0.16 kg/m^2^ higher (95% CI 0.12, 0.20 kg/m^2^) at 8 years). Children of mothers with medium educational level on average had a higher BMI from the age of 18 months onwards compared to children with high-educated mothers (e.g. 0.02 kg/m^2^ higher (95% CI 0.00, 0.04 kg/m^2^) at 18 months and 0.07 kg/m^2^ higher (95% CI 0.04, 0.11 kg/m^2^) at 8 years) (Fig. 3, Supplementary Table S4).

Paternal educational level was inversely associated with their children’s BMI from the age of 1 month to 8 years (Fig. 3). Children with low-educated fathers on average had a higher BMI from the age of 1 month to 8 years compared to their peers with fathers with high education (e.g. 0.04 kg/m^2^ higher (95% CI 0.02, 0.06) at the age of 1 month and 0.21 kg/m^2^ higher (95% CI 0.17, 0.24) at the age of 8 years). Children of fathers with medium educational level on average had a higher BMI from the age of 4 years to 8 years compared to their peers with high-educated fathers (e.g. 0.03 kg/m^2^ higher (95% CI 0.01, 0.06) at 4 years and 0.07 kg/m^2^ higher (95% CI 0.03, 0.11) at 8 years) (Fig. 3, Supplementary Table S4).

Maternal income was inversely associated with a children’s BMI from the age of 18 months onwards (Fig. 3). Children with below average income mothers on average had a higher BMI from the age of 1 year onwards compared to their peers with mothers who earned greater than /equal to mean income (e.g. 0.02 kg/m^2^ higher (95% CI 0.00, 0.04) at 18 months and 0.06 kg/m^2^ higher (95% CI 0.03, 0.10) at 8 years) (Fig. 3, Supplementary Table S4).

Paternal income was inversely associated with children’s BMI from the age of 9 months onwards (Fig. 3). Children of fathers who earned below mean income on average had a higher BMI than their counterparts with fathers who earned greater than /equal to mean income (e.g. 0.02 kg/m^2^ higher (95% CI 0.00, 0.03) at 9 months and 0.09 kg/m^2^ higher (95% CI 0.06, 0.12) at 8 years) (Fig. 3, Supplementary Table S4).

Results of interaction analyses revealed interaction effects at some age points in the association between SEP indicators and weight and BMI. For example, children of low educated mothers weighed more from the age of 18 months onwards compared to their peers with higher educated mothers, irrespective of maternal income. The same was found for paternal education but with an earlier onset, from 12 months onwards. Within low-income mothers, children of low educated mothers had a higher BMI growth trajectory up to 8 years compared to their peers with mothers with higher education. The same was found within mothers who earned greater than/equal to mean income but with a later onset, from 12 months onwards. Irrespective of paternal income status, children of low educated fathers had a higher BMI growth trajectory from the age of 1 month to 8 years compared to their peers of high-educated fathers (Supplementary Table S5).

## Discussion

This study showed that there were socioeconomic differences in weight, height, and BMI trajectories among Norwegian children from the age of 1 month to 8 years, although the magnitudes of the differences were generally small. Maternal and paternal educational differences in children’s weight and BMI trajectories emerged during infancy, continuing to age 8 years. Maternal and paternal educational differences in children’s height were not found. Parental income-related differences in children’s weight trajectory emerged at the age of 1 month and continued up to 4 years for maternal and up to 1 year for paternal income-related differences. Small parental income-related differences in children’s height emerged at the age of 1 month, continuing to 8 years. Parental income-related differences in children’s BMI emerged early (18 months and 9 months for maternal and paternal income, respectively) and continued up to 8 years. Interactions between education and income were found at some age points.

Previous studies with data from birth demonstrated that socioeconomic inequalities in children’s BMI emerged from 1 month continuing to 5 years in France^20^; from 4 to 10 years in England^21^; from 1 to 7 years in Denmark^24^, and from 3 years to 4.5 years in the Netherlands^3^. The age at which socioeconomic inequalities in children’s BMI trajectory emerged in our study were earlier than in studies from England^21^, Denmark^24^ and the Netherlands^3^, but consistent with results from a French cohort study^20^. The differences in the age at which these inequalities emerged might support the evidence that early life factors such as breast feeding, smoking during pregnancy, dietary and physical activity behaviours, which affect BMI, have different socioeconomic patterns in different countries^41–42^. Our study results indicated that Norwegian children showed the same pattern of socioeconomic inequalities in OW/OB trajectories as other high-income countries showing a higher proportion of childhood OW/OB among the lower socioeconomic groups^43,44^, which is in accordance with the reversal hypothesis^45^.

An early onset of socioeconomic inequalities in children’s BMI was found in this study. Interventions against these inequalities targeted early in life are thus needed. Furthermore, body weight change is caused by an interaction of factors. Thus, this early onset of socioeconomic inequalities and widening with increasing age might be due to differences in exposures to factors influencing child weight development at different stages of development. In this regard, studies have documented socioeconomic differences in the factors influencing child weight development such as prenatal factors^46^, breast-feeding^12^, complementary feeding practices^13,14^, childhood dietary behaviours^15–18^, and sedentary behaviours^19^. However, the extent to which these factors contribute to the occurrence and continued widening of socioeconomic inequalities in children’s weight development are not fully understood^47^; such studies are thus warranted.

Investigating how socioeconomic inequalities in children’s weight and height growth trajectories evolve with increasing age is important to understand the roots of the inequalities in BMI or OW/OB among children. Our findings demonstrated that both parental income and education related inequalities in children’s weight trajectory emerged early in infancy and only parental education-related inequalities were evident after early infancy. The socioeconomic inequalities in the children’s height growth trajectories were observed for parental income only. The SEP indicator specific results in the inequalities in children’s weight and height trajectories might indicate that parental education-related differences in BMI among Norwegian children could be attributed to educational related differences in children’s weight, rather than height. Meanwhile, income-related differences in children’s BMI could be attributed to differences in children’s height and weight up to the infancy stage and to differences in children’s height afterward.

We found that children with low educated parents had higher weight gain velocity at most of the age points studied. Weight gain velocity is a measure of children’s growth, which reflects weight gain over a given period^48,49^. Studies have shown that children who gain weight rapidly during early life are at higher risk of obesity in later life^50^ and had a higher likelihood of developing cardiovascular diseases in early adulthood^51,52^. Thus, initiating interventions targeting inequalities in children’s weight development and growth outcomes early in life could be a critical time to break the cycle for the risk of chronic disease inequalities in later life.

### SEP indicator specific differences in the weight, height, BMI trajectories

Studies addressing socioeconomic inequalities in children’s weight, height BMI, and OW/OB have used different SEP indicators interchangeably^1,21,26,28^. In our study, we assessed the independent effects of maternal income, maternal educational level, paternal income and paternal educational level on children’s height, weight, weight gain velocity, and BMI trajectories, with results indicating that there were SEP indicator-specific associations in children’s weight, height and BMI trajectories among Norwegian children aged 1 month to 8 years. In general, differences between maternal and paternal SEP indicators were not very significant, in terms of both timing and magnitude, although some differences were observed. Our results showed that the effect estimates in the association between parental income and education with children’s weight, weight gain velocity, and BMI were higher for the parental education related differences than for parental income related ones. This implies that parental educational status contributes more for the inequalities in children’s weight and BMI trajectories than parental income status. Results from interaction analyses of parental education and income in their association with children’s weight and BMI showed that despite similar income levels, those children with higher parental education had more favorable weight and BMI trajectories at some age points, showing the additional protective impact of education. Our finding supports by the finding by a study conducted in Germany highlighting the particular importance of parental education in its association with obesity over the other socioeconomic position indicators used^53^. The protective effects of a higher parental education on favorable children’s weight development might be because of the impact of education on parents’ ability to process health information leading to improved health-related decisions in parenting practice. This might in turn affect their motivation to adopt a healthy lifestyle, and act as role models for their children^27,54,55^. Thus, considering different parental SEP indicators in future studies exploring socioeconomic inequalities in child weight development is encouraged.

### Strengths and limitations

The strengths of this study include the large sample size, the prospective assessments of weight, length/ height over a long follow-up period and the assessment of the independent effects of SEP indicators on trajectories of child weight, height and BMI. In addition, the missing anthropometric measurements were handled with the use of a growth model, an approach that can overcome the problem of loss to follow-up, which tend to be more pronounced among the less advantaged and less healthy population^57–58^.

However, this study has weaknesses as well. The anthropometric measurements of the children were not based on direct measurements; they were based on parental reported measurements (although consultation of the children’s health card was suggested for some age points), which might introduce measurement errors. The cohort includes only those who read Norwegian; as a result, ethnic differences in the existing socioeconomic inequalities could not be explored. Self-selection in MoBa is also a concern. Women participating in MoBa are older, better educated and include fewer smokers than the general population of pregnant women^59^. Therefore, effect estimates for the socioeconomic differences in children’s weight, height, and BMI in the current study are likely underestimated; the generally small magnitude of differences between groups should thus be seen in light of this potential underestimation.

## Conclusions

Socioeconomic differences in children’s weight, height and BMI trajectories emerge early in infancy and remain up to 8 years of age. The magnitude of these differences varied by indicator of socioeconomic position. Early interventions to combat these inequalities are thus needed to avoid or minimize their negative health consequences. To inform such interventions, identifying the underlying mechanisms behind the early occurrence and continuing widening of the inequalities in children’s BMI with an increasing age is vital.

## Supplementary Information

Supplementary Information.

## Data Availability

The datasets analyzed during the current study are not publicly available due to data protection regulations. Researchers who want access to data sets for replication should submit an application to datatilgang@fhi.no. Access to data sets requires approval from The Regional Committee for Medical and Health Research Ethics in Norway and an agreement with MoBa.
